# Supplementary material for: Influence of Different Vegetation Types on Soil Physicochemical Parameters and Fungal Communities
Source: Microorganisms. 2022 Apr 16;10(4):829. doi: 10.3390/microorganisms10040829 (PMC9026879; doi:10.3390/microorganisms10040829)
Supplement: Supplementary file 1 [file microorganisms-10-00829-s001.zip › microorganisms-1625020-supplementary.pdf]

Supplementary Files

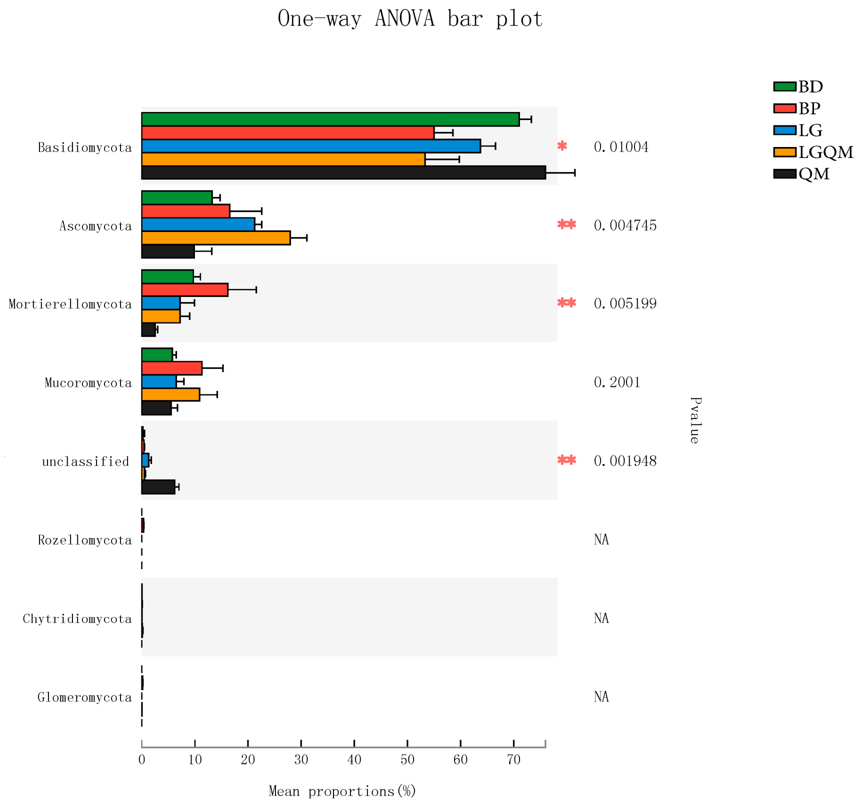

Figure S1. one-way ANOVA of the relative abundances of fungal phyla in different revegetation types.

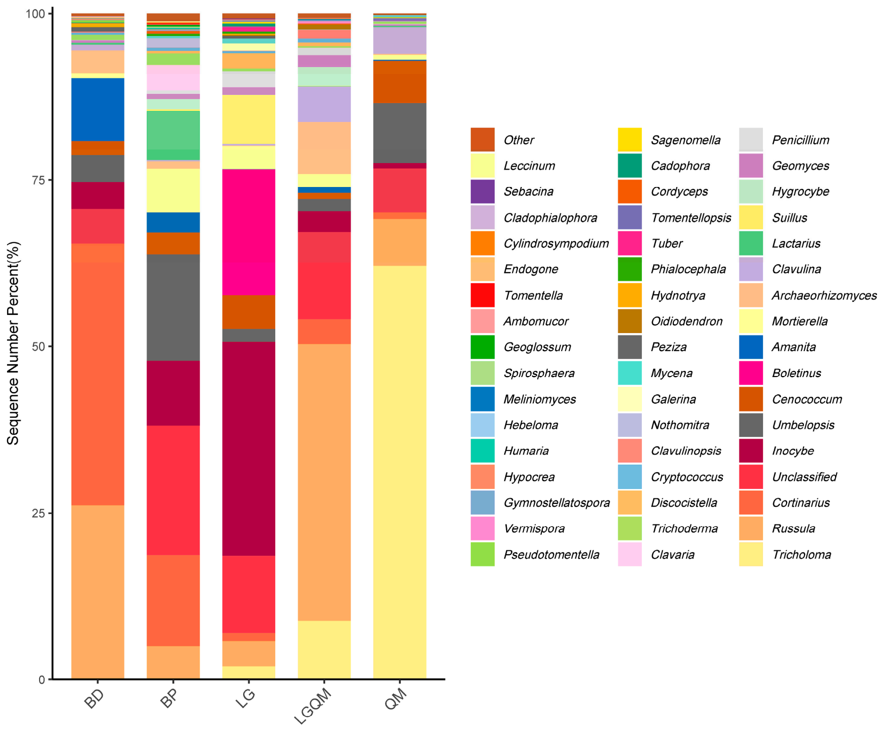

Figure S2. Relative abundance of fungal genera in five different revegetation types.

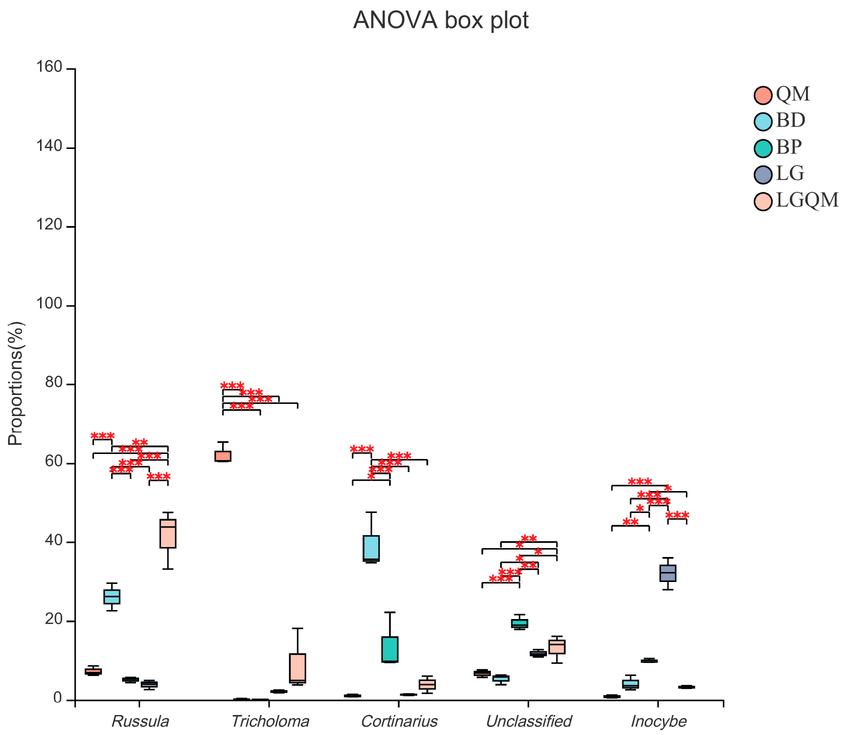

Figure S3. one-way ANOVA of the relative abundances of fungal genera in different revegetation types.
